# Promoting community health collaboration between CTSA programs and Cooperative Extension to advance rural health equity: Insights from a national Un-Meeting

**DOI:** 10.1017/cts.2020.13

**Published:** 2020-02-13

**Authors:** Michael S. Gutter, LaToya J. O’Neal, Roberta Riportella, Laura Sugarwala, John Mathias, Melissa J. Vilaro, Samantha R. Paige, Sarah M. Szurek, Giselle Navarro, Claire Baralt, Robert Rhyne

**Affiliations:** 1 Extension Administration, University of Florida, Gainesville, FL, USA; 2 Department of Family, Youth and Community Sciences, University of Florida, Gainesville, FL, USA; 3 College of Public Health and Human Sciences, Extension Family and Community Health Program, Oregon State University, Corvallis, OR, USA; 4 Center for Community Health and Prevention, University of Rochester, Rochester, NY, USA; 5 College of Social Work, Florida State University, Tallahassee, FL, USA; 6 STEM Translational Communication Center, University of Florida, Gainesville, FL, USA; 7 Department of Health Outcomes and Biomedical Informatics, University of Florida, Gainesville, FL, USA; 8 UF Extension Administration, University of Florida, Gainesville, FL, USA; 9 Clinical and Translational Science Institute, University of Florida, Gainesville, FL, USA; 10 Department of Family and Community Medicine, University of New Mexico, Albuquerque, NM, USA

**Keywords:** Rural, disparities, collaboration, cooperative extension, multilevel intervention

## Abstract

Addressing rural health disparities has unique challenges that require cross-sector collaborations to address social determinants of health and help those in need to get connected to care continuum. We brought the Clinical and Translational Science Award, Institutional Development Award Program Infrastructure for Clinical and Translational Research, and Cooperative Extension System Programs together for a one-day semi-structured meeting to discuss collaborative opportunities to address rural health disparities. Session notes and event materials were analyzed for themes to facilitate collaboration such as defining rural, critical issues, and organizational strengths in support of collaboration. Across 16 sessions, there were 26 broad topics of discussion. The most frequent topics included “barriers and challenges,” “strategies and opportunities,” and “defining rural.” There is a growing understanding of the opportunity that collaboration between these large programs provides in addressing rural health disparities.

## Introduction

Addressing health disparities remains a national priority, with federal initiatives dedicated to reducing preventable morbidity and premature mortality in rural communities throughout the USA [1]. Collaborative cross-sector partnerships are foundational to addressing rural health disparities. Social determinants of health, including higher rates of poverty and limited healthcare access in rural communities, challenge health promotion specialists and researchers to fill the chasm that perpetuates these rural–urban health disparities [2,3]. Community and societal change requires action from policy and healthcare system stakeholders; however, coordinating these networks and facilitating their sustainability remain a concern in healthcare service and policy [4]. This paper describes the power of facilitating collaborations between the Cooperative Extension System (CES) and translational research networks supported by the National Institutes of Health (NIH) to mobilize partnerships and to address systemic factors that perpetuate rural health disparities in the nation.

### Background

#### Clinical and translational science award and institutional development award program infrastructure for clinical and translational research programs

The NIH created the Clinical and Translational Science Award (CTSA) program in 2006 as a key component of the NIH Roadmap for Biomedical Research [5]. Designed to shorten the time it takes for scientific discoveries to improve health, the CTSA program has been led and funded by the NIH’s National Center for Advancing Translational Sciences (NCATS) since the center’s creation in 2012. NCATS focuses on developing, demonstrating, and disseminating advances across the full spectrum of translational science “to get more treatments to more patients more quickly” [6]. The CTSA program supports a national network of approximately 60 CTSA hubs that put that approach into practice. Based at medical research institutions across the USA, each CTSA hub engages a variety of partners, collaborators, and stakeholders to advance translational research.

As part of the program model that NCATS implemented in 2014, each CTSA hub supports 11 required functions considered to be foundational for advancing clinical and translational research, as well as up to 2 optional functions based on a hub’s unique strengths or needs. CTSA hubs also support a required career development core for early-stage investigators and an optional training core for pre- and post-doctoral trainees. Of the 11 required functions, three are overarching and high-priority functions: informatics, multidisciplinary collaboration and team science, and community engagement. Another required function is addressing the needs of special populations, which include children, older adults, and people affected by health disparities, among other groups. In 2019, NCATS published a notice emphasizing the CTSA program’s interest in expanding efforts to improve rural health outcomes and eliminate health disparities, such as projects that address translational science barriers or those designed to implement approaches or training targeting rural health outcomes [7]. This might include improving access to clinical trials for rural communities, leveraging community organizations, telehealth, and other approaches

While the CTSA program has a broad national reach, not every state has a CTSA hub. Thus, in 2019, NCATS encouraged collaboration among CTSA hubs and Institutional Development Award Program Infrastructure for Clinical and Translational Research (IDeA-CTR) networks. Historically, IDeA-CTRs are based in states with low levels of NIH funding, often amid large rural areas. Their funding by NIH propels their mission with an approach that complements the CTSA program. The IDeA-CTR program supports 11 networks spanning 15 states.

#### Cooperative extension system

The CES operates as the main purveyor for outreach of the US land grant universities. Established in 1914 [8], the strengths of CES lie in its longevity, mission, and reach. In its 100 years of existence, CES has transformed the scientific landscape resulting in the development of the current US agricultural system. The CES mission of translating research into practice is achieved by bringing scientific discoveries made at universities to communities through its nationwide network of county educators and offices that serve all 3000+ counties in the USA.

CES research and outreach is supported by competitive grants processed through the research arm of the US Department of Agriculture (USDA) – the National Institute for Food and Agriculture, federal designations through the Lever-Smith Act of 1914, and through federal, state, and county partnerships. In its most basic form, CES outreach occurs through a network of university faculty (also known as state extension specialists) who are discipline experts and county-based educators (also known as county agents/faculty) who deliver that expertise locally.

Within CES, there are many national committees working to respond to emerging needs and an infrastructure of a national Extension Committee on Organization and Policy (ECOP) with several operating boards. This in particular gives extension strong foundational support to respond to emerging needs and disseminate best practices, especially in rural communities. For example, in 2014, ECOP commissioned a report that concluded that extension should do for health in its second hundred years what it did for agriculture in its first one hundred. This built upon a growing recognition that extension needed to support not only the nutritional needs of US residents, something it has been doing through USDA’s Supplemental Nutrition Assistance Program Education (SNAP-Ed), but other health needs as well. Several workgroups have been developed to address health literacy, health insurance, chronic disease, youth development, and health in all policies. Moreover, new extension specialist positions were developed at institutions across the country to support health and wellness initiatives. There are probably no comparable systems to the CES that can generate the research and quickly disseminate the resources and tools needed to all corners of the USA.

To achieve the *Healthy People 2020* goal of eliminating rural health disparities [1], CES efforts have been designed to utilize existing infrastructures that have the potential for the greatest population-level impact. CES has a long history of translating research into action to address some of the most challenging issues faced by rural America. The CTSA and IDeA-CTR programs focus on the translation of evidence-based interventions to improve patient care and population health. Leveraging the community and clinical expertise of these entities by facilitating collaboration throughout the USA may increase the reach of evidence-based solutions to eliminate health disparities.

### Purpose

Collaborative efforts that engage expertise across translational research networks and CES have significant potential to address determinants that drive rural health disparities across the nation. The federal government has demonstrated that town hall meetings and conferences bringing together key stakeholders in healthcare services delivery are effective to establish public health and policy goals that address health priorities requiring multilevel solutions. We used a similar approach in 2019 to cultivate networks between CTSA, IDeA-CTR, and Cooperative Extension Programs by hosting an “Un-Meeting,” where attendees from different disciplines and fields shared experiences and generated innovative solutions in an unstructured conference environment. At our Un-Meeting, we convened rural health stakeholders from across the nation to explore how these sectors could work together to rapidly and efficiently achieve national goals related to alleviating rural health disparities. We invited agencies whose mission it is to address rural health needs and created a forum for them to share their understanding of rurality and the disparities that characterize rural healthcare contexts. In addition, the need to collaborate in addressing these issues warranted the discussion about how collaboration might work and the challenges that might arise from doing so.

The purpose of this short communication is to describe our process for cultivating partnerships for translating research, practice, and policy. We included topics such as barriers and strategies for multilevel collaborations and initiatives, as well as participants’ local understandings of “rurality.” Finally, we highlight how extension and using a Health Extension Framework is poised to promote community health collaborations to advance rural health equity.

## Methods

### Un-Meeting Process

The University of Florida Institute of Food and Agricultural Sciences Extension (UF/IFAS Extension) in partnership with the University of Florida Clinical and Translational Science Institute (CTSI) applied for and received funding from the national CTSA program coordinating center at the University of Rochester to host an Un-Meeting on Rural Health and Health Equity (https://www.ctsi.ufl.edu/ctsa-consortium-projects/an-un-meeting-on-rural-health-and-health-equity/). In advance of the event, attendees were invited to share descriptions of their expertise, resources, and a few words or an image that described what rural means to them. Responses alluded to participants’ multifaceted understandings of rurality and included both physical (e.g., “country,” “agriculture,” “isolation”) and community culture (e.g., “self-reliance,” “family orientation,” “traditional”) features.

The overall format of the Un-Meeting started with morning and afternoon “lightning talks,” or 4-minute presentations from experts in rural health and equity. After each set of presentations, attendees were asked to write a topic (keyword, question, etc.) that they would like to discuss on a piece of paper that was collected and compiled for immediate review. Meeting organizers categorized the responses, which were used to identify discussion session topics through processes of face validity. Attendees then chose to participate in session topics of interest to them. Prior to the meeting, faculty and staff with PhD or community engagement expertise volunteered to facilitate each session. In the spirit of the Un-Meeting format, which allows attendees to drive the agenda and discussion, the role of the facilitators was to be supportive, but not directive, in helping to catalyze conversation. Trainees and staff with experience in qualitative data collection were paired with each facilitator to serve as note-takers and to capture main ideas and thoughts during the break-out discussions. The number of participants in each session varied, and participants were able to attend multiple sessions.

There was one facilitator and one note-taker in each 40-minute session, which operated as an open forum. Some note-takers transcribed the discussions close to verbatim, whereas others described the key points of the discussion with bullet-pointed lists, sentences, and paragraphs. The purpose of these sessions was to obtain information from stakeholders and disseminate the results to inform practice, research, and policy; therefore, the sessions were not audio-recorded, nor transcribed verbatim, but instead analyzed with a time-intensive qualitative approach. Rapid qualitative analyses, such as the approach taken by our Un-Meeting team, have been shown to be a reliable method compared to results from in-depth analyses provided by stakeholders [9].

### Data Analysis

Two PhD trained researchers analyzed the data from each session. Coder 1 completed an initial read-through of the notes and applied open, descriptive categories to the text. A second round was conducted with Coder 2 to confirm existing and identify additional descriptive categories in session notes. A final review of topics was conducted by both coders. Process notes were kept during coding to capture key decisions and insights that were used to inform final analysis and interpretation [10]. A heat map of frequently discussed topics was created to indicate frequency of topics per session. Codes were not mutually exclusive. In the next phase of data analysis with session notes, coders extracted raw data pertaining to the emergent theme “defining rural.” This included comments that questioned existing or proposing new definitions of rurality. These data were analyzed in depth to describe desires surrounding updating the concept of rurality. A word cloud was created using word frequencies of the data extracted where word size corresponds to the frequency with which that term was mentioned.

## Results

The Un-Meeting was held in Gainesville, Florida, and was attended by 119 stakeholders from 30 states. Attendees were primarily from CTSA (39%) and Cooperative Extension (29%) organizations, followed by other university researchers (13%), government agencies (8%), non-profits and citizen scientists (7%), and IDeA-CTR sites (4%). The majority of representation was from the southern region of the nation (64%), with nearly equal attendance from western (11%), north central (15%), and north east (10%) regions.

### Thematic Analysis of Discussion Sessions Generated by the 4 × 4 Presentations

**Fig. 1.**
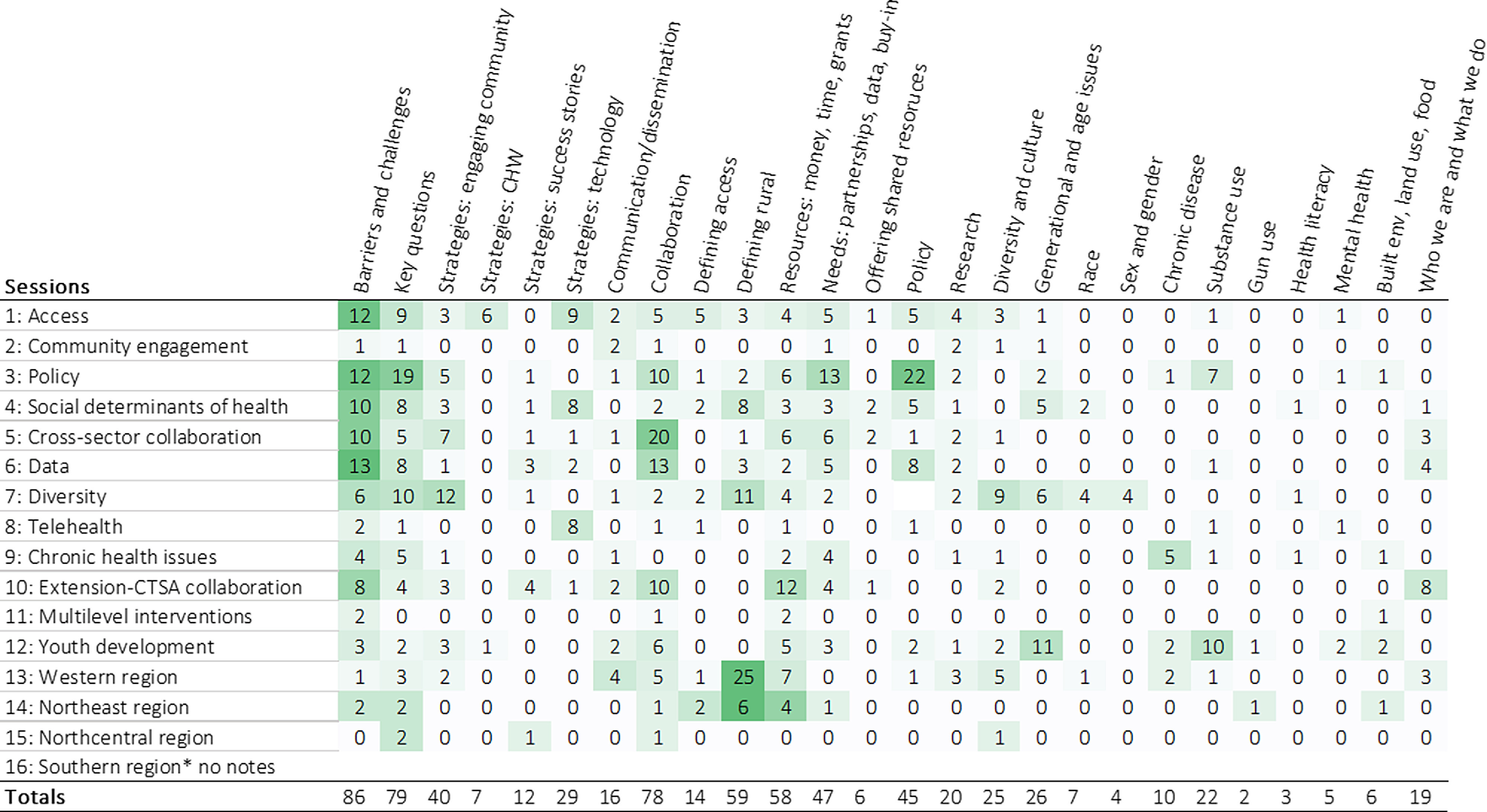
Heat map of frequently discussed topics across sessions. Interpretations of topic frequency may be more meaningful within a session than across sessions, due to variations in structure of notes.

Across 16 sessions, there were 26 broad topics of discussion (emergent themes). The heat map provides an overview of the general topics discussed in each session (Fig. 1). Across sessions, the most frequent topics of discussion were “barriers and challenges,” “strategies,” and “defining rural.” Notably, participants expressed a desire for updated definitions of rurality to facilitate research, community engagement, cross-sector collaboration, and overall to facilitate meeting the health needs of rural populations. We will discuss these frequent topics next.

### Barriers and Challenges to Promoting Health in Rural Populations

Nearly all sessions included discussions about barriers and challenges to promoting rural health equity. These included challenges of existing economic models and the untapped potential of existing resources (e.g., funding, expertise). These themes were most prominent in the sessions on data, access, policy, social determinants of health, and collaboration.

#### Barrier 1: Economic models underfund solutions

Attendees discussed the challenges of economic structures contributing to insufficient funding for grants and initiatives to tackle complicated issues. Funding models were described as not equipped to address multifactor, multisector issues that influence the health of rural populations. Business approaches to healthcare were discussed as ineffective, with sentiments indicating competitive marketplaces might not be conducive for effectively addressing rural health disparities. For example, attendees described that local agencies report limited support in paying for sustainable evidence-based solutions. Organizational and individual financial issues were viewed as a barrier to accessing preventive and primary health services among rural populations.

#### Barrier 2: Untapped potential of existing resources

Disconnects in the network of stakeholders and limitations of untapped, existing resources were barriers commonly mentioned across sessions. Attendees discussed two limitations: not knowing what other people or organizations could offer to aid in shared goals and the lamented disconnect in their network of stakeholders. Specifically, there was a lack of knowledge regarding access to specific resources such as services, expertise, data, or people. Attendees also noted the role of CES as a university- and county-level resource and the underutilization of extension agents’ expertise as partners for health promotion. Other resources mentioned included SNAP-Ed, paraprofessionals, communication, and translation experts for dissemination.

### Strategies and Opportunities for Promoting Health in Rural Populations

Fig. 1 shows the four specific strategies to promote rural health equity: engaging community, community health workers (CHWs), and improving technology.

#### Strategy 1: Community engagement

Community engagement was mentioned 40 times across 10 sessions, and CHWs were noted 7 times across two sessions. There was an emphasis on prioritizing community engagement using a system-level approach. Elements of community engagement included having bi-directional collaborations and sustained engagement. Attendees suggested setting up structures and systems to support long-term, sustainable community engagement as opposed to one-time interactions. Strategies consistent with a system-centered approach to community engagement included formal training and curriculum, ongoing engagement, collaboration, and dissemination. This approach is consistent with the CES model and processes.

#### Strategy 2: CHWs

A need for sustained engagement with community also implied funding as an essential part of the relationship. Attendees discussed that when a community perceives engagement as sustainable, it generates trust. Thus, proactive, early engagement is a strategy to facilitate community trust and success. Talk of CHWs was present in some sessions with attendees recognizing their critical role as community liaisons, while acknowledging the importance of prioritizing a system to support community efforts.

#### Strategy 3: Technology

Attendees discussed supplementing face-to-face healthcare interactions with technology (e.g. telehealth, apps, eHealth education modules, texting) as a scalable, low-cost strategy to address challenges such as limited funding. This includes understanding technological affordances that are accepted among priority audiences in rural communities, as well as the types and features of technology that are ultimately effective in promoting the health of rural adults. This strategy further recognizes that the various types of relationships technology can facilitate such as patient–provider; provider–provider; and CHW–provider. Strategies to ensure this occurs include enhancing the digital skillset of rural communities and strengthening the perceived usefulness of electronic health technology and online resources.

### Beyond the Zip Code: Conceptualizing and Measuring Rurality to Address Needs

Participants were asked in advance of the meeting to share words they associated with rural communities, and the word cloud results included in one of the Un-Meeting 4 × 4 presentations illustrated the multiple layers of rurality – including its “place,” the “geography,” and “social identity” of the “community culture” – that rural health initiatives should aim to address. Rural communities are traditionally underserved, and with the rising number of racial/ethnic minorities and immigrants relocating to rural regions, access to culturally adapted and competent services remains problematic. This Un-Meeting further validated the call to extend our conceptualization of rurality beyond the technical definitions of Rural-Urban Continuum Area Codes and to consider the lived experiences, unique community capital, and diversity of residents across rural communities.

**Fig. 2.**
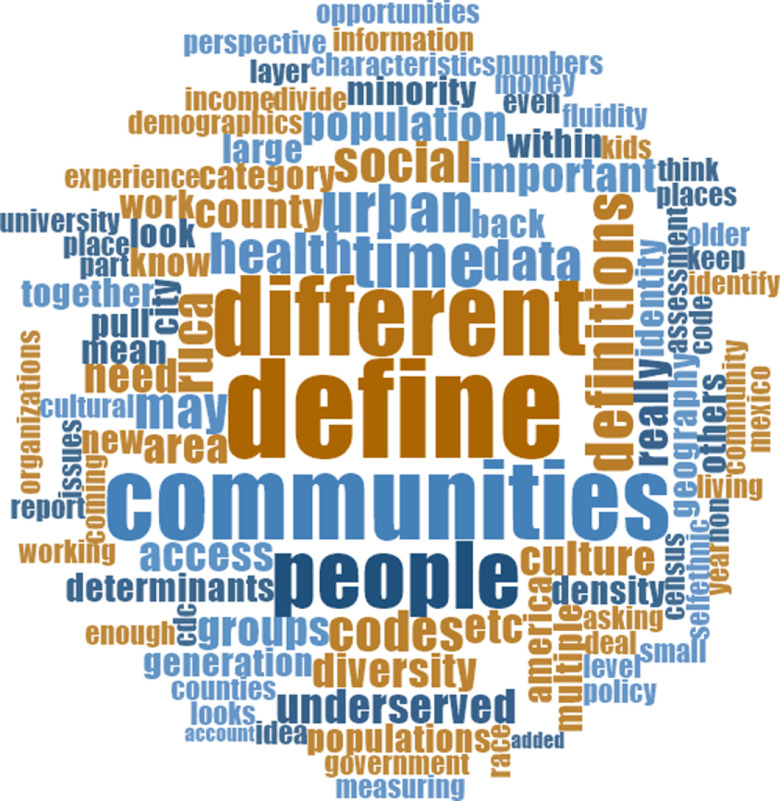
Word cloud of “defining rural” session notes.

Rural communities have needs, values, and opportunities that differ from those of urban communities. Fig. 2 shows the word cloud that captures aspects of rural communities and rural health challenges. Establishing equal opportunities and resources without adaptation to the unique features of rurality may inadvertently contribute to inequities. This possibility further suggests a need to create an updated understanding of rurality. That is, without collaborating with local community partners to reach consensus on definitions and measures, it will be challenging to manage and monitor efforts to determine program effectiveness and sustainability.

## Discussion

The Rural Un-Meeting was designed to bring together stakeholders with rural and health equity expertise to foster cross-sector collaborations. The participatory methods generated both session topics and main themes within those sessions that align with the CTSA priority functions of informatics (e.g., data, telehealth, and other strategies for communicating via technology), collaboration and team science (e.g., cross-sector collaborations, multilevel interventions), and community engagement (e.g., tapping existing resources, community “buy-in,” employing CHWs). Importantly, the Un-Meeting participants recognized the need to adapt evidence-based solutions for health equity using a multidimensional definition of rurality. Translating research into action is especially key for historically underserved rural populations, and Cooperative Extension Services infrastructure can serve as a liaison between rural communities and universities. This section will discuss specific issues raised at the Un-Meeting.

### Opportunities for Working With Extension

Extension should be considered as an ideal asset for university partners, including their CTSAs, and health science centers that are undertaking the work of eliminating rural health disparities. Many of the barriers and challenges as well as the strategies for addressing rural health disparities described by meeting participants provide support for facilitating effective collaboration between Cooperative Extension, CTSA, and IDeA-CTR programs. The local county extension offices, often owned in partnership with the county government, are the front doors to their respective Land Grant Universities. Extension faculty, educators, and agents are well positioned to help address social determinants of health that challenge rural communities such as food access, economic development, and environmental concerns. Many hold faculty appointments on their parent campuses with expectations to provide direct education (e.g., courses on chronic disease reduction or how to access health care) and address barriers to change through policy, systems, and environmental interventions. These are all done to further the goals of promoting positive behavior, systemic change, or technology transfer.

Importantly, these extension personnel (whether located on campus or locally in a county) have a reputation of being unbiased, trusted collaborators in their communities. They are experienced with community-engaged work where community members are seen as necessary parts of the process and can help less experienced researchers incorporate this style into their work. This social capital, and the general expertise of extension faculty, can add value to translational science initiatives. We have found two collaborative efforts particularly helpful for providing examples of successful partnership.

## Examples of Effective Collaboration

### Extension Health Partnership

Meaningful community integration powered by institutional collaborative forces is largely responsible for tackling some of the nation’s major public health issues. As an example, Health and Human Sciences extension at Purdue, in partnership with the nursing school, sought to design a program that covers dietary planning, clinical guidelines on cholesterol, and blood pressure, as well as ways to monitor heart health [11]. Through Community Health Partnerships [12], Purdue Extension collaborates with the Indiana Clinical and Translational Science Institute to prioritize the health needs of vulnerable populations using an assembly of community health coalitions. CheP is a network that connects prominent community figures and university faculty to engage program participants in research, provide pilot funding for community-based health studies, as well as training and further networking among stakeholders [13]. Engaging communities in both the research and solving community challenges to health is an important theme for both CTSA and CES programs.

This example helps highlight how community-based projects that leveraged the CES relationship and being focused in the work being done together.

### Health Extension Framework

The Health Extension Framework is another model worth mentioning in this context, particularly for centers considering creating similar connections to communities beyond what CES can provide. Partly in response to the funding of new ideas for addressing health disparities incorporated into the Affordable Care Act, Kaufman developed the Health Extension Framework modeled on Cooperative Extension’s outreach modality to address the social determinants of health and limited healthcare resources in rural settings [12]. This model uses Health Extension Regional Offices (HEROs) to undergird newly formed community health networks linking rural areas and university medical centers across New Mexico. The HEROs are able to vocalize and localize the most urgent health needs of their communities, serve as intermediaries to recruit a healthcare workforce, increase health literacy, and introduce the latest medical research and innovation. These gaps that HEROs fill within their communities mirror the strategies and opportunities discussed throughout the Rural Un-Meeting.

To support the critical role HEROs play in addressing barriers to healthcare access, the University of New Mexico Clinical and Translational Science Center (CTSC) assists in research funding, training opportunities, and community engagement. Upon implementation and dissemination, resources are allocated to study the HERO model itself with respect to the particular needs of a community. Resources are also used for the development of informative promotional tools to increase the number of stakeholders and local partnerships who are invested in improving health conditions through advocacy and policy. In the New Mexico model, researchers, HEROs, CES, and university clinics work together to implement strategies designed to combat prevalent rural health issues. For example, a researcher who was experiencing recruitment challenges for a study on teenagers and obesity sought the help of a local HERO, who was connected through the University of New Mexico (UNM) Health Sciences Center (HSC) Clinical and Translational Science Center (CTSC) office [14]. The HERO was able to assist the researcher by introducing her to school leaders and parents, as well as explain the relevance of her role in conducting a study that was aimed at reducing obesity in young adults residing in their county. Additionally, a HERO was called to work alongside the department of psychiatry at UNMHSC to prepare training sessions in Mental Health First Aid for “first responders” to help relieve barriers in accessing counseling or therapy [9].These examples illustrate how a similar model could be undertaken to address the health-specific topics from the Un-Meeting sessions, such as chronic disease, substance use, gun use, health literacy, and intergenerational and aging issues.

The HEROs and CTSC collaboration through a Health Extension model serves as a successful framework in addressing the health concerns of rural populations in New Mexico. Five grants have been funded through NIH, Agency for Healthcare Research and Quality, and CTSC totaling $7,409,002, allowing for greater expansion in the delivery of resources and medical services as well as community engagement work to overcome barriers to the quality of care in rural underserved communities [15]. This is an example of how the issue of sustainability and grants, raised at the Un-Meeting, can be addressed. These two networks have aligned interests for reducing health disparities in rural communities. They also have a strong shared capacity and a historical record of successful interventions.

### Conclusion

As we consider the potential for collaborations between CES and CTSAs, we offer the following practical considerations. When exploring partnerships for rural health equity work, it is important for all parties to establish shared expectations and processes. If our goal is to help reduce disparities in rural communities, building a foundation of understanding between those in CTSAs, IDeA-CTR, and CES is a critical first step in this process. If one is intending to work in a rural community, engaging extension at the outset is key to identifying needs and conceptualizing projects that take into account the local context. Ideal collaborations involve joint efforts to understand the needs of the community by engaging medical and extension specialists along with the community in research and discovery. These lead to collaborative implementation strategies that can help address issues such as prevention of chronic disease, addressing social determinants of health, and learning how to manage age-related conditions which can include cancer.

Furthermore, dissemination throughout the research and implementation process is a vital part of these collaborations. Extension voices can be tapped to share opportunities to participate in research; they can also be used to share research findings and more importantly, design educational programs to incorporate the latest research findings to change behavior. Extension faculty can help identify communication channels that might work best within certain rural communities. The ability to replicate studies or scale up a project is strong when partnering with wide-reaching CES systems that have the capacity to tap into rural areas. CES systems are in every state and most counties, including rural counties.

Finally, advocating to support collaborations between CES and CTSA or IDeA-CTR programs is an important activity. There are numerous agencies that fund projects related to rural communities and their health issues. As their advocates, we can find requests for proposals, for example, written in ways that encourage and indeed prioritize those collaborations. As we have argued above, such collaborations have great promise for addressing rural health disparities. While there are examples of successful collaborations between the two groups, there remains much unrealized potential.
